# Predictive accuracy of wideband absorbance in children with large vestibular aqueduct syndrome: A single-center retrospective study

**DOI:** 10.1016/j.heliyon.2024.e33776

**Published:** 2024-06-27

**Authors:** Wen Jiang, Xuanyi Li, Yi Mu, Huiying Zhang, Naveena Konduru, Yuehua Qiao, Fei Zhao, Wen Liu

**Affiliations:** a Department of Otolaryngology, The Affiliated Hospital of Xuzhou Medical University, Xuzhou, 221000, China; b The Second College of Clinical Medicine, Xuzhou Medical University, Xuzhou, 221000, China; c Auditory Engineering Laboratory of Jiangsu Province, Xuzhou, 221000, China; d The College of Medical Technology, Xuzhou Medical University, Xuzhou, 221000, China; e Centre for SLT and Hearing Sciences, Cardiff School of Sport and Health Sciences, Cardiff Metropolitan University, Cardiff, CF5 2YB, UK, Wales

**Keywords:** Wideband absorbance, LVAS, AUC, Children, Retrospective case-control study

## Abstract

**Objectives:**

This study aimed to assess the clinical significance of Wideband Absorbance (WBA) in children with Large Vestibular Aqueduct Syndrome (LVAS), which could potentially serve as diagnostic and predictive markers for LVAS in children.

**Design:**

This was a single-center retrospective case-control study. Audiological measurements and Wideband Acoustic Immittance (WAI) were performed. Propensity score matching (PSM) was considered to treat group imbalance. The Receiver Operating Characteristic (ROC) curves and area under the ROC curve (AUC) were used to evaluate the sensitivity and specificity of WBA.

**Study sample:**

Participants included 42 children with LVAS and 163 normal children aged 6 months −11 years recruited from clinical audiology settings between 2019 and 2021.

**Results:**

The WBA at Tympanometric Peak Pressure (WBA_TPP_) and Ambient Pressure (WBA_A_) in the LVAS group were significantly lower than those of the control group at 1259–2000 Hz but higher at 4000–6349 Hz (*p* < 0.05, power >0.8). The WBA_A (1587 Hz)_ AUC value was 0.805, identifying a score ≤0.565 as indicative of a LVAS risk.

**Conclusions:**

WBA holds promise in distinguishing LVAS from the normal condition and warrants further exploration as a tool to examine the influence of inner ear pressure on acoustic energy transmission in the middle ear.

## 1 Introduction

Large vestibular aqueduct syndrome (LVAS) is a prevalent congenital anomaly of the inner ear that results in early-onset Sensorineural Hearing Loss (SNHL) [[1], [2], [3], [4]]. LVAS accounts for approximately 13–15 % of pediatric SNHL cases [[5], [6], [7]]. The majority of LVAS cases (81–94 %) show bilateral SNHL, which can be fluctuating and/or progressive, and is frequently accompanied by vestibular impairment, such as episodic vertigo or postural instability [1,8]. Factors that elevate intracranial pressure, such as viral infections, head trauma, or emotional stress, can precipitate sudden hearing loss and/or vertigo in LVAS patients [9].

The vestibular aqueduct (VA) is a narrow bony channel in the temporal bone that links the medial wall of the vestibule to the petrous part of the temporal bone. It contains a membranous labyrinth that communicates with the endolymphatic sac, which modulates endolymph metabolism. LVAS is diagnosed by radiological techniques (e.g., CT scan) according to VA size: a VA diameter of 1.5 mm or more at the operculum and 1/2 of the vestibular aperture indicates LVAS [2]. Enlarged VA may also alter cerebrospinal fluid (CSF) pressure dynamics in the inner ear [10,11]. CSF reaches the inner ear through the VA, the cochlear aqueduct, and the internal auditory canal [11]. Changes in intracranial pressure in LVAS patients can propagate to the inner ear via the enlarged VA and cause excessive cochlear pressure. This can rupture the membranous labyrinth and disrupt electrolyte balance or introduce toxic or hyperosmolar fluid into endolymph [[12], [13], [14]]. Increased endolymphatic pressure can also compress blood vessels in the inner ear and reduce blood flow, impairing hair cell function [15].

Although endolymphatic reflux induced by elevated intracranial pressure due to head trauma or intracranial infection may explain the sudden hearing fluctuations in LVAS patients [12,16], the relationship between intracranial pressure and inner ear pressure is complex and poorly understood. Nakashima et al. [15] found low-frequency conductive hearing loss in LVAS patients and hypothesized that increased inner ear pressure pushed the stapes inward and limited its movement. However, they did not observe any changes in the acoustic reflex as expected in early otosclerosis with stapes fixation [15]. This suggests that endolymphatic reflux affects stapes mobility differently from stapes fixation in acoustic reflex [12]. Moreover, children with LVAS older than six months often have a normal tympanogram but show ‘Meniere-like’ audiometric features with significant low-frequency ABGs [17].

Such findings suggest the existence of a “third window” conductive hearing loss, specifically associated with inner ear lesions [17,18]. Building upon this, Reynard et al. [19] classified LVAS into one of two categories: either intralabyrinthine third mobile window abnormalities (TMWA) or TMWA-like conditions, a distinction based on anatomical and radiological assessments of inner ear structures. TMWA itself manifests as a conductive hearing loss triggered by inner ear abnormalities, such as an enlarged vestibular aqueduct syndrome (LVAS) or a dehiscent superior semicircular canal (SSC) [19]. Additionally, a correlation has been established between larger VA dimensions and increased ABGs [20]. Despite these advances, the exact mechanism responsible for TMWA-like conductive hearing loss in LVAS remains an open question, meriting further investigation.

Merchant et al. [21] proposed acoustic reflexes, vestibular evoked myogenic potential (VEMP), distortion product otoacoustic emission (DPOAE), and laser doppler vibrometry (LDV) as potential diagnostic tests for tympanic membrane abnormalities, but their validity and reliability need further verification. MRI/CT are commonly used to diagnose third window lesions [19], but they have limitations such as high cost and risk of radiation and sedation, especially for children under two years old [22]. Therefore, alternative methods are needed to reduce these drawbacks. TMWA patients exhibit air-bone gap and hearing loss confined to frequencies below 2000 Hz [21], but it is unclear how common this phenomenon is in other diseases that affect hearing. Conventional acoustic admittance with a single frequency of 226 Hz or 1000 Hz cannot accurately reflect inner ear pressure changes, which may affect the diagnosis of TMWA.

Furthermore, acoustic reflex in TMWA is less sensitive than in patients with stapes fixation [15], and thus it may not be a reliable indicator of TMWA. VEMP has been suggested for diagnosing vestibular diseases and third window lesions, but it may not be feasible for patients with severe Tullio phenomenon or limited neck movement [23]. Abnormal DPOAE and air-bone gaps of pure-tone audiometry have been reported in animal experiments with third window lesion [24], but there is a lack of clinical evidence to support this finding. In addition, although LDV is a sensitive measurement of inner ear pressure, it has limited clinical application, and umbo velocity measurement is mainly used for research purposes [25]. Thus, there is a gap in the literature on an effective test for diagnosing LVAS.

Recent evidence has suggested that Wideband Acoustic Immittance (WAI) could be a sensitive method to detect the changes in inner ear pressure, which is hypothesized to be increased in LVAS [26]. WAI using frequencies from 226 to 8000 Hz has been proposed to be more sensitive to changes in both mass and stiffness components and to detect minor changes in the transmission characteristics [[26], [27], [28], [29], [30], [31]]. Several studies have investigated the use of WAI in inner ear diseases, such as Meniere disease [27,32], superior semicircular canal dehiscence [27,32], inner ear malformations [29], and LVAS [26]. Wideband absorbance (WBA), a primary parameter from the WAI measurement, reflects the efficiency of the middle ear in absorbing sound across different frequencies. The predictive accuracy of WBA in ear diseases has also been studied. Keefe et al. [33] compared normal and surgically confirmed otosclerosis and found that the AUC was 0.95 at 2800 Hz for TPP condition and 0.88 at 1400 Hz for ambient pressure condition for WBA. Karuppannan and Barman [34] also observed high diagnostic values of WBA in otosclerosis at 1000 Hz (>90 % sensitivity and specificity). Aithal et al. [30] evaluated normal and surgically confirmed otitis media with effusion and found that the highest AUCs were for WBA at ambient pressure with the frequency of 1500 Hz (0.92), WBA at tympanometric peak pressure with the frequency of 1250 Hz (0.91).

Zhang et al. [26] performed a pilot study of WBA in 13 children with LVAS and compared them with normal children. They observed that the WBA of children with LVAS was markedly lower than normal children at middle frequencies (1000–2000 Hz) and at specific ambient and peak pressures [26]. This suggests that WAI could be a useful and non-invasive tool for assessing inner ear pressure. However, the reliability of this evidence is limited by the small sample size and the lack of age- and gender-matched controls. Our study aimed to explore the WBA characteristics of children with LVAS with age and gender matched controls and the predictive accuracy of WBA in ears with LVAS confirmed by temporal bone CT scans.

## 2 Materials and methods

### 2.1 Ethics and consent

This retrospective study received approval from the Ethics Committee of the Affiliated Hospital of Xuzhou Medical University (Approval No. XYFY2021-KL133-01), ensuring adherence to Good Clinical Practice and the principles of the Declaration of Helsinki, emphasizing respect for individual confidentiality and including a waiver for informed consent.

### 2.2 Clinical procedures

This single-center investigation gathered data from the medical record system of the Affiliated Hospital of Xuzhou Medical University, with data collection and compilation undertaken by two seasoned researchers. The research team reviewed medical records for patients identified per the study protocol to gather information on demographics, audiological assessments and image results. Patient identifiers were systematically generated using hospitalization numbers to integrate data across various databases, assigning unique data identifiers to each patient. Upon completion of data coding, identifiers were removed to preserve confidentiality before statistical analysis.

We adopted a multi-faceted approach to rule out middle ear pathologies that could potentially influence WBA values. This approach included physical examinations, traditional 226-Hz tympanometry, comprehensive hearing assessments, and CT scans (administered to the LVAS group only). Comprehensive audiological assessments were completed prior to the WAI tests. The study protocol adhered to STROBE guidelines.

### 2.3 Study population

The LVAS group was composed of 42 children (aged 6 months to 11 years) identified from clinical audiology records between January 2019 and December 2021. These cases were diagnosed with Large Vestibular Aqueduct Syndrome (LVAS) as per medical records during this period. Children who failed newborn hearing screening or whose parents reported hearing loss were considered. Inclusion criteria were as follows: no instances of newly reported hearing and balance issues within one month at the time of WAI measurement; Otoscopy with clean external auditory canal and healthy tympanic membrane; Hearing loss measured using auditory steady-state response and auditory brainstem response with/without acoustically evoked short latency negative response (ASNR) or pure tone audiometry (or play audiometry) for children older than three years old; 226 Hz tympanogram with a type A and the volume of the external auditory canal within the normal range, defined as within −100 to +50 daPa and peak compliance range from 0.3 to 0.9 ml; CT results with the large vestibular aqueduct, ≥1.5 mm, bilaterally, and no other ear malformation.

The control group was comprised 163 normal-hearing children (aged 6 months to 11 years) who had undergone audiological evaluations in the same period. All participants were comprehensively examined to confirm as normal hearing. Inclusion criteria were as follows: No significant history or abnormal findings present about middle-ear pathology and no aural symptoms with one month; Otoscopy with clean external auditory canal and healthy tympanic membrane; For children older than 3 years, pure tone audiometry (or play audiometry) within the normal range (≤20 dB HL bilaterally between 250 and 8000 Hz) and for children 6 months to 3 years, a pass result of distortion product otoacoustic emission (60 % frequencies or more showing DP ≥ −5 dB and SNR ≥6 dB) and auditory brainstem response within the normal range (≤20 dB nHL, click stimuli); 226 Hz tympanogram with a type A and the volume of the external auditory canal within the normal range, defined as within −100 to +50 daPa and peak compliance range from 0.3 to 0.9 ml.

### 2.4 Wideband acoustic immittance assessment

Utilizing the Interacoustics IMP440 system (Interacoustics, Denmark), we determined WAI over a 226–8000 Hz frequency range and a −300 to +200 daPa pressure range. The software provided detailed metrics for absorbance, reflectance, and impedance at each distinct frequency and pressure. The Titan device provides a value of the resonance frequency (RF) after completing WAI measurements. According to the instruction provided by Interacoustics (2023), the rationale for measuring RF using Titan is to identify the lowest frequency at which susceptance (B) reaches zero mmhos. This occurrence results from cancellation between the stiffness and mass elements of susceptance (B) at this particular frequency. This suggests that RF represents the optimal point for the efficient transfer of sound energy within the middle ear system.

To ensure reliable results, we used silicone-based probe tips of varying sizes to ensure an optimal ear canal seal. Testing commenced only after a system-generated seal check signal confirmed no air leakage. Tests were administered under controlled, quiet conditions for our pediatric subjects. Guardians received prior instruction on maintaining a disturbance-free environment by minimizing child behaviors that might affect the results, such as crying or yawning. Any deviations from these guidelines necessitated test repetition to ensure accurate data collection. Each test lasted around 2 min per ear. The RF was automatically generated and recorded by the Titan device to identify the lowest frequency at which the value of B equals zero mmhos. Static compliance (SC) was derived from the maximum absorbance at the frequency point of 226 Hz. Both WBA at tympanometric peak pressure (WBA_TPP_) and WBA at Ambient Pressure (WBA_A_) measurements were used in our study.

### 2.5 Statistical analysis

The tests yielded data that was analyzed based on the following parameters.

1) Characteristics of 1/3 octave-averaged WBA against frequency. Notably, measurements were taken both at tympanometric peak pressure (TPP) and ambient pressure (A).
2) The static compliance (SC) derived from the WAI tests at the frequency of 226 Hz.
3) The resonance frequency (RF) ascertained from the WAI tests.
4) Participants' demographics, specifically their age and gender.

Baseline characteristics including the age and gender were compared between children with LVAS and with normal hearing using a *t*-test or Mann-Whitney *U* test for continuous variables and chi-square tests for categorical variables. Descriptive statistics included mean (standard deviation), median (interquartile range), or n (%). In order to minimize selection bias and control for clinically relevant variables, propensity score matching (PSM) was considered for baseline characteristics [35]. A one-to-one matched analysis was then performed. An absolute standardized mean difference *d* < 0.1 in PSM indicated a negligible difference between groups [35].

A repeated-measures Analysis of Variance (ANOVA) was employed to analyze data for ears with and without LVAS. To adjust for any violations in sphericity and compound symmetry, the Greenhouse-Geisser correction was implemented. The factors selected for between-group analysis included group (control versus LVAS) through a range of frequencies at WBA_A_ or WBA_TPP_ condition. Independent samples *t* tests were applied to explore differences in WBA values across the LVAS and control groups. Statistical significance was defined as *p* values with the sequentially rejective Bonferroni test (Bonferroni-Holm procedure) (Abdi, 2010) correction for multiple testing less than 0.05. Independent samples *t*-tests were also employed to compare SC and RF. Sample size was conducted by power analysis with value more than 0.8 as sufficient. Receiver-operating characteristic (ROC) curve analysis was performed to compare the diagnostic value of WBA at frequencies, RF, and SC to LVAS. The area under the ROC curve (AUC) was calculated. The optimal cut-off value was identified according to the Youden index. AUC values between 0.5 and 0.7 had low accuracy, AUC values > 0.7 had a mild diagnostic value, and AUC values above 0.9 had high accuracy [36]. For analyses we used Statistical software: SPSS 26.0 (IBM SPSS Statistics), Origin 2023 (OriginLab INC.) and G. power 3.1 (University of Düsseldorf, Germany).

## 3 Results

### 3.1 Population characteristics

In this study, we recruited children aged 6 months to 11 years and carefully selected a suitable control group, as detailed in Table 1. Initial analyses revealed a clear imbalance between the two groups regarding baseline factors of age and gender. The age data did not conform to a normal distribution, as indicated by the Shapiro-Wilk test (*p* < 0.001). Consequently, we employed the Mann-Whitney *U* test to assess age differences between the groups, which yielded a significant result (*p* < 0.05). Gender homogeneity across the two groups was evaluated using the Chi-square test, which showed no statistically significant difference in gender distribution (χ^2^ = 3.005; df = 1; *p* = 0.083).

**Table 1:** Baseline Covariates between the control and LVAS groups with PSM.

Baseline Covariate	Pre- Propensity- Matching		*p* value	*d*	Post- Propensity- Matching		*p* value	*d*
	LVAS Group (n = 42)	Control Group (n = 163)			LVAS Group (n = 41)	Control Group (n = 41)		
Age (yr)	4.06	2.71	0.010	0.48	4.00	4.04	0.953	0.01
Gender			0.233	0.22			0.823	0.00
Male	15 (35.71 %)	75 (46.01 %)			15 (36.59 %)	15 (36.59 %)		
Female	27 (64.29 %)	88 (53.99 %)			26 (63.41 %)	26 (63.41 %)		

**Fig. 1:**
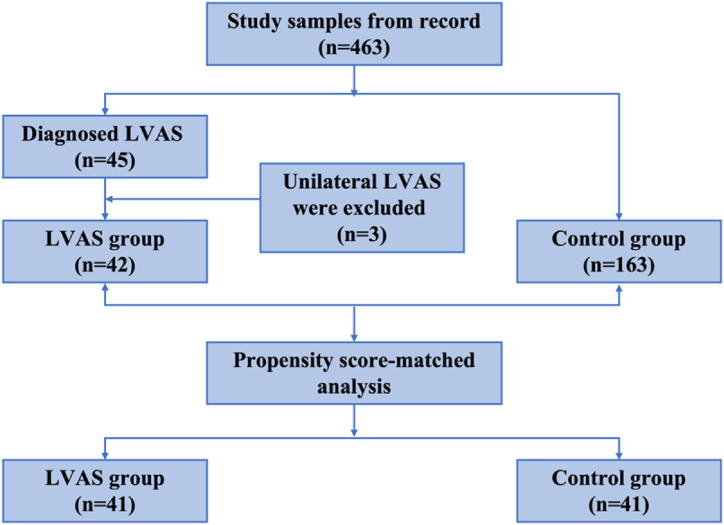
Flow chart of study population selection.

To minimize selection bias, propensity score matching (PSM) was applied to correct for the noted imbalances [35]. Each variable's absolute standardized mean difference was *d* < 0.1 after matching, indicating a balance between the groups. Post-PSM, the age-related imbalance was rectified (*p* > 0.05) and the difference between groups in two covariates was all negligible (*d* < 0.1). Consequently, 82 ears (41 participants) for each group were taken into comparison (Fig. 1). It is important to mention that the LVAS group had one fewer participant after PSM, due to the inability to find a suitable match for one individual based on the stringent criteria set forth, thus maintaining the comparability and integrity of the matched groups.

All matched LVAS childrenwere bilaterally confirmed by CT and showed moderate to profound hearing loss with a type A tympanogram and demographic information and audiological characteristics was shown in Table 2.

**Table 2:** Demographic information and audiological characteristics of the children with LVAS.

Participant NO.	Age (Year/Month)	Gender	ASSR-R^a^	ASSR-L^a^
P1	5Y	F	91.25	72.50
P2	7Y	M	56.00	40.00
P3	8Y	M	96.00	72.00
P4	7Y	M	R100.00	100.00
P5	5Y	M	112.50	110.00
P6	11Y	M	R100.00	100.00
P7	4Y	M	98.75	97.50
P8	4Y	M	115.00	103.75
P9	7Y	M	108.75	86.25
P10	4Y	F	75.00	56.25
P11	4Y	F	100.00	98.75
P12	5Y	F	111.25	58.75
P13	7Y	M	111.25	112.50
P14	4Y	M	113.75	105.00
P15	5Y	M	51.00	59.00
P16	3Y	F	90.00	93.75
P17	5Y	F	80.00	67.50
P18	3Y	F	96.25	100.00
P19	4Y	F	85.00	110.00
P20	10Y	M	92.50	81.25
P21	5Y	M	93.75	108.75
P22	10Y	M	62.50	58.75
P23	7Y	F	78.75	62.50
P24	8Y	M	88.75	78.75
P25	3Y	M	97.50	62.50
P26	6 M	M	L97.50	97.50
P27	1Y5M	M	103.75	97.50
P28	1Y9M	M	93.75	90.00
P29	1Y1M	M	100.00	100.00
P30	6 M	M	95.00	93.75
P31	6 M	F	53.75	55.00
P32	1Y9M	F	75.00	66.25
P33	6 M	F	65.00	57.50
P34	2Y7M	M	97.50	103.75
P35	8 M	F	62.50	67.50
P36	2Y2M	M	97.50	85.00
P37	6 M	F	91.25	91.25
P38	7 M	M	88.75	92.50
P39	7 M	F	78.00	92.00
P40	2Y	M	82.50	92.50
P41	6 M	M	97.50	91.25

a : ASSR average threshold at 500,1000, 2000, 4000 Hz by air conduction. R: right ear. L: left ear.

### 3.2 Wideband absorbance (WBA) in children aged 6 months-11 years between the LVAS and control groups

In an analysis employing a repeated measures ANOVA with WBA_TPP_ as the dependent variable, a significant main effect was observed for group (control versus LVAS) [F (1, 162) = 8.657, *p* = 0.004, η^2^ = 0.051]. Additionally, the main effect of frequency [F (3.099, 502.052) = 343.915, *p* < 0.001, η^2^ = 0.680], and the interaction between group and frequency [F (3.099, 502.052) = 13.279, *p* < 0.001, η^2^ = 0.076] were notably significant.

In a repeated measures ANOVA with WBA_A_ as the dependent variable, and group and frequency as independent variables, the analysis of WBA_A_ as the dependent variable yielded no significant main effect for group (control versus LVAS) [F (1, 162) = 3.809, *p* = 0.053, η^2^ = 0.023]. However, the data showed a significant main effect for frequency [F (2.947, 477.431) = 318.917, *p* < 0.001, η^2^ = 0.663] alongside a significant interaction between group and frequency [F (2.947, 477.431) = 16.450, *p* < 0.001, η^2^ = 0.456].

**Fig. 2:**
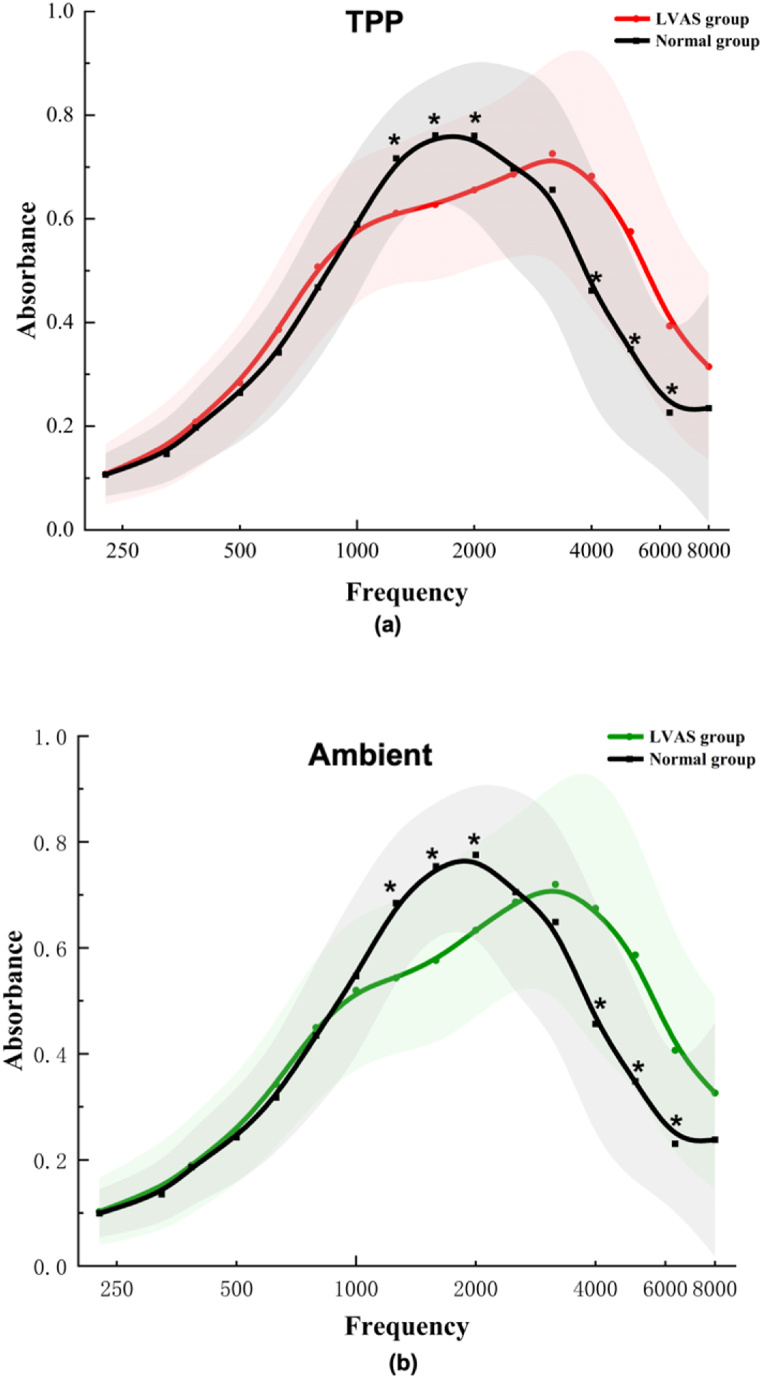
Mean (±standard deviation of the mean) absorbance at TPP (a) and ambient (b). * Statistically significant differences of the absorbance at individual frequencies between LVAS group and normal group. The shaded region indicates the range of mean ± 1 standard deviation (SD) across the frequencies. between 226 and 8000 Hz.

Analysis of WBA in children aged between 6 months and 11 years showed significant differences between the LVAS and control groups. Specifically, both the WBA_TPP_ and WBA_A_ values for the LVAS group were significantly lower than those of the control group at frequencies 1259–2000 Hz, while higher at frequencies 4000–6349 Hz (*p* < 0.05), and all the power values were more significant than 0.8, showing statistical test effectiveness (Table 3, Fig. 2a&b).

**Table 3:** Comparison of WBA_TPP_ and WBA_A_ between the control and LVAS group.

Frequency (Hz)	WBA_TPP_			WBA_A_		
	Control (N = 82)	LVAS (N = 82)	*Power*	Control (N = 82)	LVAS (N = 82)	*Power*
226	0.107	0.108	0.064	0.099	0.103	0.119
324	0.147	0.159	0.279	0.135	0.147	0.282
385	0.197	0.208	0.209	0.187	0.190	0.079
500	0.265	0.283	0.303	0.243	0.256	0.219
629	0.342	0.387	0.75	0.318	0.343	0.409
793	0.468	0.508	0.545	0.435	0.450	0.171
1000	0.590	0.583	0.092	0.547	0.520	0.301
1259	0.717	0.611^a^	0.999	0.684	0.543^a^	0.999
1587	0.761	0.628^a^	0.999	0.754	0.576^a^	1.000
2000	0.760	0.656^a^	0.998	0.776	0.633^a^	0.999
2519	0.698	0.686	0.112	0.706	0.686	0.176
3174	0.656	0.726	0.72	0.649	0.720	0.722
4000	0.462	0.682^a^	0.999	0.457	0.674^a^	0.999
5039	0.349	0.575^a^	0.999	0.348	0.587^a^	1.000
6349	0.226	0.393^a^	0.999	0.231	0.406^a^	0.999
8000	0.235	0.315	0.816	0.238	0.326	0.874
Low frequencies (226–793 Hz)	0.254	0.276	0.875	0.236	0.248	0.295
Middle frequencies (1000–2519 Hz)	0.705	0.633^a^	0.999	0.693	0.592^a^	1.000
High frequencies (3174–8000 Hz)	0.386	0.538^a^	0.999	0.385	0.543^a^	1.000

The categories ‘low frequencies (226–793 Hz)', ‘middle frequencies (1000–2519 Hz)', and ‘high frequencies (3174–8000 Hz)' represent the averaged values of the wideband Absorbance at ambient pressure (WBA_A_) and Wideband Absorbance at tympanometric peak pressure (WBA_TPP_) measurements across the defined frequency ranges.

a *p values with Bonferroni-Holm (BH) correction for multiple testing were less than 0.05*.

### 3.3 Predictive accuracy of WBA in LVAS

Based on the ROC analysis results, both sensitivity and specificity were assessed. Among children aged 6 months-11 years, the WBA_TPP_ showcased the highest diagnostic potential for LVAS, registering a cut-off value of 0.317 at 6349 Hz (sensitivity, 68 %; specificity, 82 %). In contrast, the WBA_A_ had a cut-off value of 0.565 at 1587 Hz (sensitivity, 57 %; specificity, 91 %). The AUCs of combined frequencies were considered as middle frequencies (MF, 1000–2519 Hz) and high frequencies (HF, 3174–8000 Hz), which were all below 0.8. Detailed values can be found in Table 4.

**Table 4:** The area under the receiver operating curve (AUC) and 95 % CI, sensitivity, and specificity for WBA_TPP_, WBA_A_, RF, and SC.

Group	Frequency (Hz)	AUC	95 % CI	Cutoff	Sensitivity	Specificity
WBA_TPP_	1259	0.725	0.650–0.792	0.710	80.49	58.54
	1587	0.747	0.674–0.812	0.601	50.00	91.46
	2000	0.700	0.623–0.769	0.724	70.73	67.07
	4000	0.746	0.672–0.811	0.753	53.66	90.24
	5039	0.766	0.694–0.829	0.514	69.51	81.71
	6349	0.767	0.694–0.829	0.317	68.29	81.71
WBA_A_	1259	0.761	0.688–0.824	0.631	74.39	68.29
	1587	0.805	0.736–0.863	0.565	57.32	91.46
	2000	0.748	0.674–0.812	0.782	80.49	58.54
	4000	0.736	0.661–0.801	0.681	62.20	82.93
	5039	0.776	0.704–0.837	0.492	70.73	78.05
	6349	0.775	0.704–0.837	0.337	64.63	89.02
WBA_TPP-MF_	1000–2519	0.680	0.603–0.751	0.691	68.29	60.98
WBA_TPP-HF_	3174–8000	0.763	0.690–0.826	0.552	58.54	87.80
WBA_A-MF_	1000–2519	0.730	0.655–0.796	0.634	64.63	74.39
WBA_A-HF_	3174–8000	0.766	0.693–0.828	0.550	60.98	87.80
RF	–	0.650	0.566–0.735	814.5	61.60	70.00
SC	–	0.371	0.283–0.458	0.265	63.90	36.10

The categories ‘MF (middle frequencies, 1000–2519 Hz)', and ‘HF (high frequencies, 3174–8000 Hz)' represent the averaged values of the wideband Absorbance at ambient (WBA_A_) and Wideband Absorbance at Tympanometric Peak Pressure (WBA_TPP_) measurements across the defined frequency ranges. RF: resonance frequency. SC: static compliance.

**Fig. 3:**
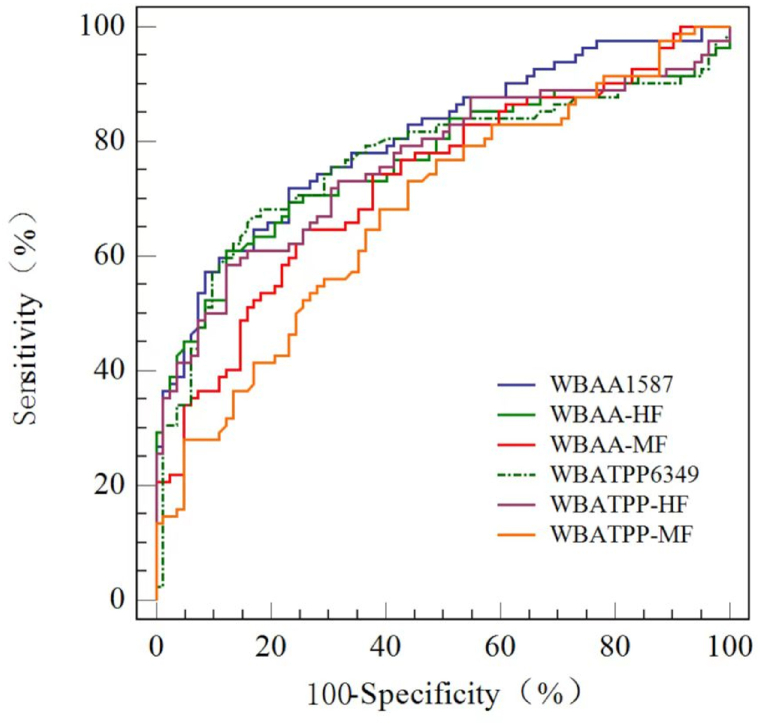
ROC curves to compare the ability of WBA_A_ and WBA_TPP_ to discriminate LVAS from normality.

Children with LVAS exhibited differences in several parameters compared to the control group. The average RF of 737.63 Hz (SD: 252.53) was lower than the control group 892.22 Hz (SD: 334.91) (*p* = 0.010), with an AUC of 0.650 (95 % CI: 0.566 to 0.735). Conversely, the average SC of 0.36 ml (SD: 0.21) was higher than the control group 0.36 ml (SD: 0.21) (*p* = 0.014), with an AUC of 0.371 (95 % CI: 0.283 to 0.458). The WBA_A (1587 Hz)_ could provide the highest specificity and accuracy [AUC, 0.805, 95 % CI (0.739–0.863)] in predicting LVAS at the age of 6 months–11 years (Fig. 3).

## 4 Discussion

**Fig. 4:**
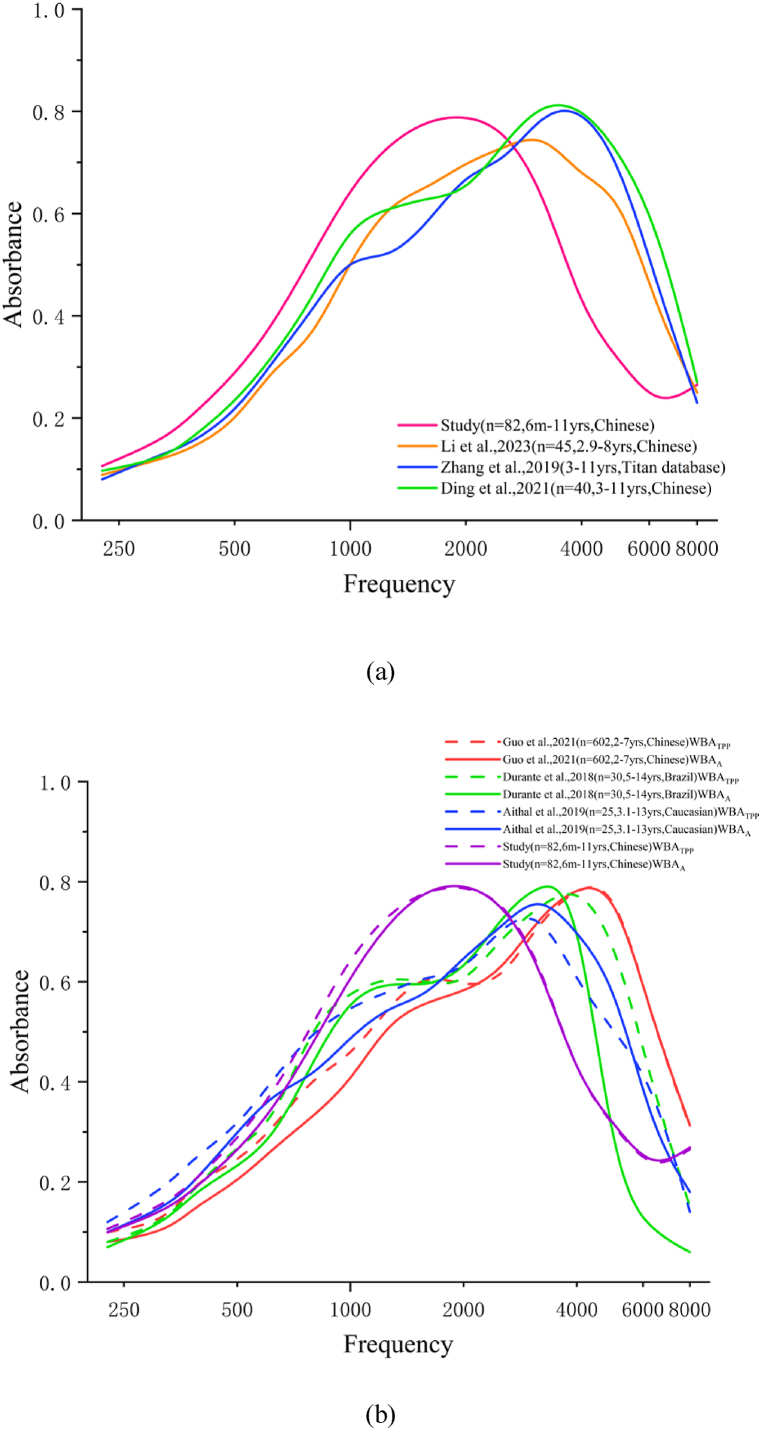
Comparison of the WBA from the control group to other reports of ‘normal’ WBA (a: LVAS studies -WBA_TPP_; b: other pediatric studies- WBA_TPP_& WBA_A_)).

We observed that WBA_TPP_ and WBA_A_ were lower in the LVAS group than in the control group at middle frequencies (1259, 1587, 2000 Hz). However, these measures were higher at high frequencies (4000, 5039, 6349 Hz). This diverges from the findings of Zhang et al. [26], who reported significantly lower WBA in children with LVAS across several frequencies. As seen in Fig. 4s a&b, age may play a role in affecting WBA measurements. In our normal pediatric sample, older age led to higher WBA values at high frequencies (Fig. 4a). There are a few factors that could contribute to this discrepancy. Firstly, the age distribution of our sample differs significantly from that in the Zhang et al. [26] study (6m-11yrs vs. 3–11yrs), which could have particular effects on high-frequency measurements. Secondly, the sample sizes between our study and Zhang's (82 vs.13) are also different, affecting the reliability and generalizability of the results. Given these variations in age distribution and sample size, direct comparisons between the two studies should be interpreted cautiously. Additionally, the power of our study was greater than 0.8 at middle and high frequencies, but not at low frequencies, suggesting that further research is required to clarify these findings at low and high frequency ranges. By integrating advanced techniques with clinical data analysis, future research may enhance the reliability of WBA for LVAS diagnosis.

A possible explanation for the decreased WBA at middle frequencies might be the enlarged endolymphatic vessels and endolymphatic bursa with the increased volume of endolymphatic fluid [18], potentially causing pathological expansion of the third window and increase the inner ear pressure [37]. The high inner ear pressure may reduce the mechanical impedance of the inner ear [15] and consequently may reduce the impedance of the stapes. However, our findings also showed differences in WBA between the LVAS and control groups under the TPP condition, even after adjusting for negative pressure, indicating that negative middle ear pressure alone doesn't account for these differences. This aligns with studies showing that negative middle ear pressure can lead to reduced WBA at mid frequencies and increased WBA at high frequencies [30].

On the other hand, the increased WBA at high frequencies could be related to the middle ear RF and standing wave. Motallebzadeh et al. [38] used a fluid-structure coupling finite element model to measure the admittance of wideband acoustic input and observed that two peaks at 5 kHz and 6.4 kHz were associated with the resonance of the middle ear cavity and standing wave. Pang et al. [39] applied a biomechanical method to investigate the effect of enlarged VA on CHL, and established a lumped-parameter model to predict the overall trend of larger ABGs at low frequencies, but with limited precision in individuals, especially at high frequencies. Therefore, while negative inner ear pressure might be part of the explanation, the exact mechanism remains unclear, and further biomechanical studies are warranted.

It has been reported that increased inner ear pressure can alter the stiffness of the tympano-ossicular conductive system [26,40]. We hypothesize that the enlarged endolymphatic vessels and endolymphatic bursa elevate the inner ear pressure, which leads to the increased mass of the middle ear and causes the air-bone gap [15]. A conductive hearing loss (CHL) caused by a third window lesion in the inner ear results in a mobile window on the scala vestibule, showing bone conduction enhancement and air conduction deterioration [17]. WBA is effective at detecting CHL caused by middle ear pathology [30,[41], [42], [43], [44], [45], [46], [47]] and it also seems sensitive to detecting the inner ear CHL [[26], [27], [28], [29]].

Understanding the relationship between inner ear pressure, mass and stiffness of the middle ear, and inner ear conductive hearing loss provides a foundation for our exploration of RF and SC. Our findings reveal statistical differences in RF and SC between the LVAS and control groups. The average RF of 892.22 Hz (SD = 334.91) in the control group aligns with Downing et al. [48] observations of age-related RF reductions: 928.95 Hz for ages 4–6 years, 872.80 Hz for ages 7–9 years, and 863.68 Hz for ages 10–13 years. RF is the frequency at which stiffness-reactance and mass-reactance are equal. If the stiffness of the middle ear increases, the resonance frequency is higher than the standard frequency. Conversely, if the mass element of the ear increases (or its stiffness decreases), the RF decreases [49]. Sato et al. [50] suggested that RF is related to the immittance of the cochlea, due to the increased inner ear pressure and the decreased compliance of the stapes. However, the diagnostic utility of RF for LVAS remains low in our study, as indicated by an AUC value less than 0.7. This is consistent with Sugasawa et al. [51] who reported low sensitivity and specificity in diagnosing Meniere's disease (41.3 % and 84.2 %, respectively). Moreover, Bilgen et al. [37] observed different RF results in LVAS patients under different hearing conditions, indicating that this may be associated with different mechanisms of LVAS. The mean RF values across different studies and methods show significant variability, indicating that RF can differ widely even among similar age groups [52]. Studies used different methods like Multifrequency Tympanometry (MFT) and Wideband Acoustic Immittance (WAI), which might contribute to the observed variability in RF values. The 90 % range in RF varies widely, pointing to a need for normative data, especially when using new methods like WAI. Some studies included a broad range of ages, making it challenging to develop age-specific normative data. Therefore, there is a pressing need for further studies on RF, especially with the use of new methods like WAI.

Our study compared the predictive accuracy of WBA_A_, WBA_TPP_, RF, and SC values, and found that the WBA_A (1587 Hz)_ showed high specificity in predicting LVAS (AUC, 0.805). Overall, the AUC for WBA is much higher than the parameter of SC at 226 Hz. As a non-invasive and quick objective audiological test, WAI seems cost-effective in the LVAS test battery, especially for children younger than two years.

Although these values indicate a promising diagnostic tool, it should be noted that the test did not achieve the highest sensitivity and specificity values. This limitation may stem from various factors such as the inherent variability in LVAS manifestations among the population, the testing protocol's constraints, or the influence of other confounding variables. Prompt consideration should be given to increasing the sample size and exploring high specificity and sensitivity through machine learning. Further research and refinement of the testing methodology may be needed to optimize sensitivity and specificity, providing a more robust and precise tool for LVAS diagnosis.

This study has several limitations that should be taken into account. Firstly, the absence of pure tone audiometry data due to the age variability among participants could impact the comprehensiveness of our findings. Secondly, despite employing Propensity Score Matching (PSM) to balance the age discrepancies across groups, the broad age range remains a limitation and due to the small sample size, we were unable to conduct subgroup-specific ROC analyses in age for WBA measures. Future studies with larger sample sizes, more refined age categorizations, and perhaps longitudinal studies might provide deeper understanding of the complex interactions involved. Additionally, we aim to explore dynamic characteristics of WAI in LVAS, such as phase variations and 3D WAI images.

## 5 Conclusions

In a pediatric cohort with Large Vestibular Aqueduct Syndrome (LVAS), we investigated wideband absorbance (WBA) patterns. Compared to controls, the LVAS group showed significantly lower WBA_TPP_ and WBA_A_ values at 1259–2000 Hz and higher values at 4000–6349 Hz. A WBA_A_ value of ≤0.565 at 1587 Hz emerged as a potential diagnostic threshold for a LVAS risk. Despite promising diagnostic utility of WBA, challenges in sensitivity, specificity, and study design advocate for continued research. Future studies with broader samples and enhanced tools will refine our understanding of LVAS and improve diagnostic accuracy.

## Funding

This work was supported by the Medical science and technology innovation project of Xuzhou Municipal Health Commission (XWKYHT20220149); the Open project of key laboratories in colleges and universities in Jiangsu Province (XZSYSKF2022005) and Postgraduate Research & Practice Innovation Program of Jiangsu Province (KYCX23_2940).

## Data availability

The original data can be requested by emailing wen.jiang@zxhmu.edu.cn.

## Additional information

No additional information is available for this paper.

## CRediT authorship contribution statement

**Wen Jiang:** Conceptualization, Data curation, Formal analysis, Methodology, Resources, Writing – original draft. **Xuanyi Li:** Formal analysis, Writing – original draft. **Yi Mu:** Data curation, Formal analysis, Funding acquisition. **Huiying Zhang:** Data curation. **Naveena Konduru:** Data curation. **Yuehua Qiao:** Conceptualization. **Fei Zhao:** Conceptualization, Writing – review & editing. **Wen Liu:** Conceptualization, Funding acquisition.

## Declaration of competing interest

The authors declare that they have no known competing financial interests or personal relationships that could have appeared to influence the work reported in this paper.
